# Adapted Home-Based Exercises in Dementia: An Exploratory Pre-post Pilot and Feasibility Study

**DOI:** 10.1177/15333175241263741

**Published:** 2024-06-14

**Authors:** Sophie Carrard, Stephan Eyer, Roger Hilfiker, Anne-Gabrielle Mittaz Hager

**Affiliations:** 1 111844 HES-SO Valais-Wallis, Sion, Switzerland; 2 30919 Hôpital Riviera Chablais, Switzerland; 3 Physiotherapie Tschopp and Hilfiker, Glis, Switzerland

**Keywords:** home-based exercise, dementia, prodromal or mild Alzheimer’s disease, feasibility, executive functions, functional mobility

## Abstract

The goals of this exploratory pre-post pilot and feasibility study (NCT04916964) were to assess the feasibility and effectiveness of an adapted Test-and-Exercise home-based exercise program on basic functional mobility and executive functions in persons with prodromal or mild Alzheimer’s disease. Participants followed an 8 week exercise program at home, once per week with a physiotherapist and twice per week with their usual caregiver or independently. Functional mobility and executive functions were assessed before and after the intervention. Feasibility criteria were recruitment opportunity, participation agreement rate, cost adequacy, and drop-out rate. Twelve participants aged 80.83 ± 4.65 years took part in the study. All the basic functional mobility measures showed small effect sizes. Concerning executive functions, 5 measures showed small to moderate effect sizes. The 4 feasibility criteria were met. A larger scale study would, however, need adaptations and prior research on the ability of this population to use touch-screen technology.

## Significance Statements

- The home-based exercise program T&E may be effective for patients suffering from AD; however, no clinical recommendations can be made based on the results of this study.
- Further large-scale study is needed to evaluate the effectiveness of T&E in the AD population.

## Introduction

Worldwide, over 55 million people suffer from dementia, with nearly 10 million new cases every year. The World Health Organization recognizes dementia as a public health priority through the endorsement of the “Global action plan on the public health response to dementia 2017-2025”.^1^ Alzheimer’s disease (AD) is the most common form of dementia and may account for 60%-70% of cases. The incidence rate of AD increases consistently with age from approximately .5% among individuals aged 65-70 years to approximately 6%-8% for individuals over 85 years.^2^

Besides cognitive and functional impairment, persons with AD are more at risk of falls than healthy older persons.^3^ Risk factors for falls are multiple and not always modifiable. In patients with cognitive impairment and dementia, common risk factors for falls are gait and balance impairments, behavioral disorders, malnutrition, adverse effects of drugs, fear of falling, neuro-cardiovascular instability (particularly orthostatic hypotension), environmental hazards, and the lack of integration of instruction.^4,5^ Balance impairments in elderly patients with AD might result rather from cognitive functions, such as attention and executive functions (EF).^6^ Furthermore, there is strong evidence regarding the association between cognitive impairment and serious fall-related injuries.^7^

Attention and EF, known as cognitive control and the supervisory attentional system, represent a wide range of active cognitive processes needed to respond appropriately to environmental stimuli.^8^ They could be defined as a set of cognitive processes that: (i) guide actions and behaviors essential to aspects of learning and everyday human performance tasks; (ii) contribute to the monitoring or regulation of such tasks; and (iii) pertain not only to the cognitive domain, but also socioemotional and behavioral domains of human performance.^9^ Patients with amnestic mild cognitive impairment (MCI), which includes AD, have problems with response inhibition, switching, and cognitive flexibility, which are included in these functions.^10^

Exercise, or balance or postural training have no effect to a large effect on balance, mobility, functional or postural performance among people suffering from different types of dementia.^11^ Exercise programs influence positively the number of falls, but have no effect on the step test or physiological profile assessment.^12^

Exercise ability is independently and inversely associated with the onset of dementia, AD, and cognitive impairments.^13^ In healthy older people, exergames and balance lower the use of the prefrontal cortex, which implies an improvement in EF and processing speed. Lowering the use of EF and attention resources might allow improved focus of attention while walking and therefore reduce the risk of falling.^14^ Even though physical activity is beneficial for people with dementia, there is a general lack of clarity regarding how physical activity interventions work, what outcomes can be expected, and what outcomes could be sought.^15^ Regular physical exercise (i) reduces vascular flow and diabetes, (ii) promotes neurogenesis, (iii) increases metabolic factors and muscle-derived myokines, and (iv) stimulates the production of neurotrophins, in particular brain-derived factor.^16,17^ Furthermore, regular exercise exerts anti-inflammatory effects and improves the brain redox status, ameliorating the pathophysiological hallmarks of AD, such as amyloid-beta deposition.^18^ Physical activity and exercise could improve cognition^19^ and might ameliorate depression and sleep disturbances in patients with AD.^20^ Exercise programs may also improve the ability to perform activities of daily living in people with dementia, but the intervention setting (home vs institutional) should be considered.^21^ Exercise may be associated with greater improvements in memory in trials including people with MCI than in trials including only people without cognitive impairments.^22^ Physical exercise may improve EF in community-dwelling older adults living with AD^23^ and in older adults with MCI.^24^

Test-and-Exercise (T&E) is a home-based exercise program provided on a tablet and is based on empowerment and self-efficacy.^25^ T&E has positive effects on physical outcomes in older adults at risk of falling without cognitive impairment.^26^

Although T&E program was only evaluated in elderly persons with no diagnosed cognitive impairment, it would be interesting to assess its effect in cognitively impaired older adults. Recruitment and ethics present several challenges in research involving people with cognitive disabilities.^27^ The aim of this exploratory pilot pre-post study was to assess the feasibility and effectiveness of an adapted T&E home-based exercise program on basic functional mobility and EF in persons with prodromal or mild AD.

## Materials and Methods

This was an exploratory before–after study. The protocol was approved by the Swiss Ethics Committee on research involving humans (2021-00714) and was registered at Clinical Trial.gov (NCT04916964). Intervention was described according to the Template for Intervention Description and Replication.^28^

### Participants and Recruitment

Participants were recruited at the Memory Center of Valais Hospital in Sierre by a local physician. The following inclusion criteria were set:

• living at home;
• having a medical diagnosis of prodromal or mild AD, which corresponds to stages .5 and 1 of the clinical dementia rating^29^ (Mini-Mental State Examination (MMSE)^30^ 18–23 & Functional Assessment STaging^31^ 4 or 5);
• being medically stable;
• being able to walk with or without an assistive device but without physical assistance from another person;
• being able to follow one-step commands.

Subjects were not eligible if they presented with severe vision or verbal impairments, any serious orthopedic condition, a major neurological or musculoskeletal comorbidity affecting their functional mobility (ie, cerebrovascular accident, Parkinson’s disease, recent orthopedic surgery), or a limiting cardiac or pulmonary condition.

All participants and their usual caregiver received an informed consent form during the visit of the principal investigator to their home to explain all the relevant information about the study. All included participants and usual caregivers signed and returned the consent form. All participants were given an identification code for data coding.

### Procedure

Before the beginning of the intervention, additional data were collected, such as symptoms of depression using the short version of the geriatric depression scale (GDS-15),^32,33^ environmental safety with the French translation of the Safety Assessment of Function and the Environment for Rehabilitation – Health Outcome Measurement and Evaluation (SAFER-HOME) third version,^34,35^ and quality of life using both the Quality Of Life questionnaire for Alzheimer’s Disease (QOL-AD)^36,37^ and the EuroQoL-five dimensions-five levels (EQ-5D-5 L).^38,39^ These data were collected by the principal investigator (AGMH) at the participants’ home.

**Figure 1.**
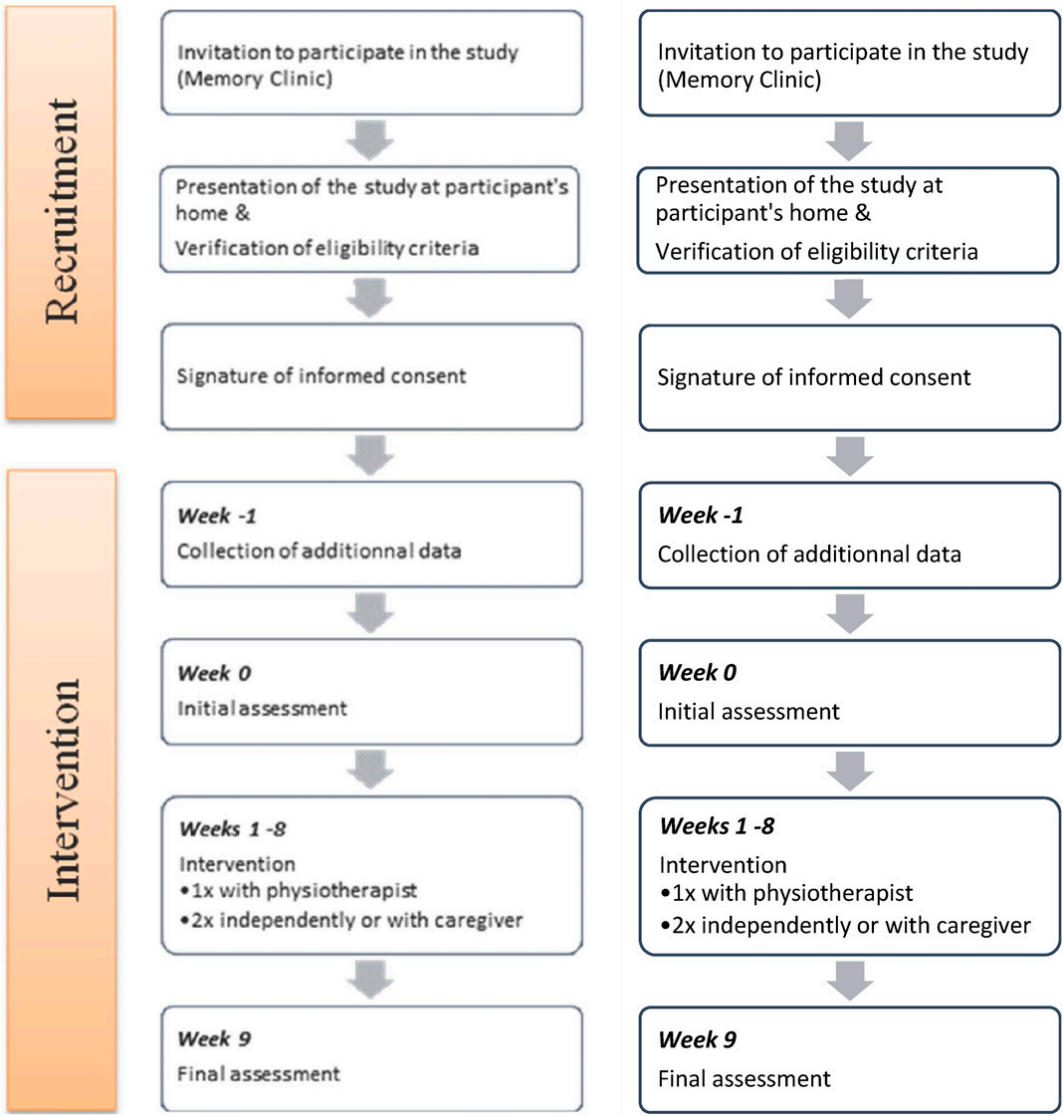
Participant’s path in the study.

The intervention lasted 8 weeks with assessments of functional mobility and EF at the beginning and end. Pre- and post-intervention assessments were performed at the participants’ homes, as performance in people suffering from AD seems to be better in a familiar environment.^40,41^ Both assessment sessions took place at the same time of day, the tests were performed in the same order, and were assessed by the same examiner. The participant’s path is shown in Figure 1.

### Intervention

The original T&E exercise home-based program was created for older adults at risk for falls without cognitive impairment. It aims to prevent falls in the older community with a program based on empowerment and self-efficacy.^25^ It is delivered on a digital tablet or in a booklet. T&E material is available contacting the last author of the current study (AGMH). The program includes 50 physical tasks (eg, forward and backward walking, slaloming between cushions, sit and stand from a chair, etc.) grouped under 14 topics related to home objects or activities (eg, the chair, on the ground, walking, etc.). Every task can be used as a test or as an exercise. When a task is tested, the persons evaluate the perceived difficulty of the task with 5 response options (0 = no difficulty; 1 = some difficulty; 2 = difficult; 3 = very difficult; 4 = too difficult). Based on the concept of self-efficacy, self-confidence, and empowerment, the participants choose a maximum of 8 exercises evaluated between zero and 2 to build their own exercise program.

The T&E program was delivered by specially trained physiotherapists. They participated to a 4-hour training, in which they were introduced to the use of the application on the tablet related to the assessment of the exercises and the setup of the patient’s program. The session took place at the participants’ homes, once a week for 8 weeks, in one-to-one sessions. The caregiver was invited to participate in the session so that both the participant and the caregiver could learn the exercises and the use of the T&E program.

For this study, participants received 8 home-based exercise sessions once a week for 2 months to activate the preserved implicit memory.^42^ Each physiotherapy session lasted between 45 and 60 minutes. The physiotherapist trained the participant and the caregiver to use the T&E exercise program on a learning-by-doing basis. The physiotherapist used the tablet for this and proceeded step by step from opening the tablet and application, to setting up the program and beginning the training. If the participant preferred not to use the tablet, the physiotherapist explained the use of the T&E program using the booklet.

Participants were recommended to train for 3 weeks using the same exercise program, once under supervision by the physiotherapist and twice under supervision by their usual or a family caregiver, or independently. The training sessions without the physiotherapist could be done at 1 time (45-60 minutes) or divided into 2 half-sessions of 25 or 30 minutes on the same day. If the chosen exercises became too easy to perform in the first 3 weeks, the participant could either increase the number of repetitions, the number of sets, or the speed of execution, as proposed in the program. After 3 weeks, the participants could choose if they wanted to continue with the same exercises or change all or some exercises while testing other tasks.

To improve adherence, the participants had to fill in weekly diaries in the training booklet. These diaries contained details of the training session: day of the week, time(s) of the day, duration(s), content of the training session (ie, which test, which exercise, etc.), remarks, falls if any, and presence of the caregiver. It was also used to plan, organize, and remember the details for the next training session. The physiotherapist checked the training booklet at each physiotherapy session, asked if something was not clear, and helped the participant to fill in the diary correctly if necessary.

After each home-based exercise session, physiotherapists used Research Electronic Data Capture (REDCap) software^43^ to fill in a description of the session, including the date, day of the week, start and end time of the session, whether the caregiver was present, how the session was conducted, and any comments that may have an impact on the participant’s follow-up.

In the case of muscle or joint pain, or the onset of any other disease, the physiotherapist took the appropriate measures, ie, adapting the exercises (sitting or lying instead of standing, decreasing the number of repetitions or series, etc.) or contacting the referring doctor.

## Outcomes

Primary and secondary outcomes were collected to assess the effect of the exercise program in older people with prodromal or mild AD. None of those outcomes has been changed during the trial. Feasibility outcomes helped to evaluate the process for a larger study.

### Primary Outcomes

Basic functional mobility was assessed with the Berg Balance Scale (BBS), the Time Up and Go Test (TUG), the 5 time sit to stand (FTSTS), and walking speed. Participants performed the TUG and the walking speed assessments 3 times. The first attempt was not evaluated. The average of the last 2 measurements were used for analysis. In both assessment sessions (initial and final evaluations), the tests were administered in the same order.

#### Berg Balance Scale

This is a valid and efficient measure of postural balance in the geriatric population and consists of 14 balance tasks, ranking in difficulty from unsupported sitting in a chair to picking up objects from the floor or turning in a circle while standing. This tool determines the patient’s ability to balance in a controlled manner during the predefined tasks. The tasks are function-oriented, and each item is scored from “0” (worst function) to “4” (best function). The scoring ranges from “0” to “56”, the higher values indicating better function. Gait is not evaluated.^44,45^ BBS has been used with older adults with cognitive deficits with excellent and test–retest reliability (Intraclass correlation (ICC) .97).^46,47^ It takes approximately 20 minutes to complete.

#### Timed Up and Go test

This assesses lower extremity function, mobility and fall risk.^48^ It is not only associated with motor performance but also with cognitive function.^49^ The task is to walk as fast as comfortable to a marker (at 3 meters), turn around, return to the chair and sit. The stopwatch starts at the command “Go”. In patients with MCI, the protocol is adapted to capture only the mobility component of the TUG and not the time required for progression of cuing strategies before the movement. The stopwatch timing begins on movement initiation, meaning when the participant’s buttocks leave the chair. In addition, the ground marker is substituted by a small orange cone. This modified protocol is specially adapted for people with AD.^50^ The TUG presents excellent test–retest reliability in both the mild (MMSE 20-28) and moderate (MMSE 10-19) cognitive impairment population (ICC .96 resp. .94).^51^

#### Five Time Sit to Stand

This is a valid assessment of dynamic balance and functional mobility in older adults and has excellent relative and absolute reliability and reproducibility.^52^ Participants begin the test sitting on an armless chair with their arms crossed over their chest and their back against the back of the chair. The assessor demonstrates the correct technique to perform the test, including coming to a full stand, defined as an upright trunk with the hips and knees extended. The instruction is, “Stand up and sit down as fast as you safely can”. Timing begins when the assessor says “GO” and stops when the participant’s buttocks touch the seat after the fifth stand.^53^ The FTSTS shows excellent interrater reliability in patient with dementia (ICC .94-.99)^54^ or with AD (ICC .79).^55^

#### Walking Speed

This is a simple, useful, and inexpensive assessment tool to measure different aspects of the ageing process.^56^ When enough space was available, the 6-meter walking test was performed. If there was any space limitation at the participant’s home, a 4-meter walking test was performed to determine the comfortable walking speed (CWS) and the maximal walking speed (MWS) in meters per second (m/s). Two meters were added prior to and following the timed portion to allow for acceleration and deceleration.^57^ (53) For assessment of CWS, participants are instructed to walk at their “usual, comfortable speed”. For MWS, the instruction is to walk as “quickly, but safely as possible without running”.

### Secondary Outcomes

Executive functions of the tripartite classification were assessed, namely, cognitive flexibility (set-shifting), updating (working memory), and inhibition.^8^ In both assessment sessions (initial and final evaluations), the tests were administered in the same order.

#### Trail marking Test Part-B (TMT-B)

This evaluates set-shifting and consists of 24 circles distributed over a sheet of paper.^58^ The circles include numbers (1-12) and letters (A–L). With a pen or pencil, the examinee must draw lines to connect the circles in an ascending pattern, with the task of alternating between the numbers and letters (1-A-2-B-3-C, etc.). The assessor demonstrated the test first to the patient using a sample sheet. The patient was instructed to connect the circles as quickly as possible without lifting the pen or the pencil from the paper. If the patient made an error, the assessor pointed it out immediately and allowed the patient to correct it.

**Figure 2.**
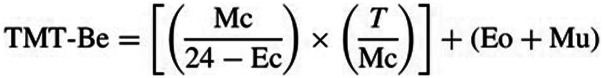
TMT-Be Score calculation (Mc: number of correct moves; Ec: commission errors; T: time; Eo: omission errors; Mu: unattempted moves).

The score is usually determined by completion time in seconds (TMT-Bs) with a maximum of 300 seconds. As people failing the task are given the highest score, the TMT-Bs masks considerable performance variability.^59^ Therefore, the score was calculated using TMT-B efficiency (TMT-Be) in this study, as it considers not only completion time but also the number of correct sequencing moves, errors of commission (ie, sequencing or tracking error, perseverative errors or proximity error^60^), errors of omission and unattempted items due to premature termination. Lower scores indicate better efficiency.^59^ The score calculation is displayed in Figure 2.

#### Digit Span Backward Test (DST-B)

This evaluates updating and is part of the Digit Span Test (DST), which consists of forward and backward recall of digit sequences. DST is part of Wechsler’s^61^ intelligence scales. DST forward (DST-F) seems to be more related to attention and DST-B to working memory.^62,63^ As per the modified protocol, participants were asked to repeat series of digits of increasing length from 2 to 8 digits in reverse order. The test includes 2 sequences of each length, and testing ceases when the patient fails to recollect any 2 sequences with the same length. The score, ranging from zero to 14, is the number of successful sequences. Higher scores indicate better updating.^64^

#### Stroop Color-Word test – Victoria (SCWT)

This assesses the ability to inhibit cognitive interference, also called response inhibition.^65^ This occurs when the processing of stimulus features affects the simultaneous processing of another attribute of the same stimulus.^65^ SCWT is validated in French using 3 panels: colors (C), words (W) and interference (I).^66^ Each panel consists of 6 lines of 4 colored stimuli, using red, yellow, blue, and green. The panels evaluate different conditions. In panel C, the participant must name the color of the dots. Panel W consists of 4 coordinating conjunctions written in color. Participants must name the color of the words. In panel I, the names of the 4 colors are written in a different color (eg, the word ‘green’ is written in red). Participants should name the color in which each word is written. The time to complete each panel is timed in seconds. The timer is not stopped when an error occurs. Two interference indices are calculated: a weak interference index (if = Time panel W/Time panel C), which evaluates inhibition capacity when the interference produced by the irrelevant response is low, and a strong interference index (IF = Time panel I/Time panel C, which evaluates inhibition capacity when the interference produced by the irrelevant response is strong.

For each panel or interference index, the score is the log10 of the time for completion or the index. For each participant, age, socio-cultural level and time for each panel was entered in a coded Excel file.^67^ The expected score is a regression with 2 predictive factors, namely age and socio-cultural level. The Z-score is the comparison between expected and participant’s scores. The performance is based on the Z-score (Z < −1.65: deficit; −1.64 to −.9: limit; −.9 to .9: moderate; .9 to 1.64: superior; ˃1.65: high superior).^66^

### Feasibility Outcomes

Feasibility was assessed considering recruitment opportunity, participation agreement rate, cost adequacy and drop out/withdrawal rate. A larger scale study is feasible when all these points are met.¨

#### Recruitment Opportunity

Memory consultations in the memory clinic of the Hôpital du Valais in Sierre occurred only once a week. Therefore, and according to the patients’ flow, the inclusion of twelve eligible subjects in the study should occur no later than 24 weeks after the start of recruitment.

#### Participation Agreement Rate

To evaluate the study as feasible at larger scale, 75% of eligible subjects should have agreed to participate in the study and signed the consent.

#### Cost Adequacy

The planned budget of the study was 90 000 Swiss Francs (CHF). It included (i) participation, ie, physiotherapy sessions and material (CHF 20 400), (ii) research staff costs (CHF 61 700) and (iii) other costs, ie, administration costs, ethical commission fee, REDCap license, etc. (CHF 8500.-). The feasibility is met if the global costs of the study were not exceeding by more than 5% the original budget, ie, CHF 94 500.-.

#### Drop Out/Withdrawal Rate

75% of the participants (9 people) should have taken part in the total duration of the study (10 weeks), as well as in the final evaluation for a larger scale study being feasible.Adverse events.

At each session, the physiotherapist asked the patient how they feel and if they encounter any health problems. The following situations were considered as adverse events (AE) and reported to the principal investigator:

• Falls that require medical attention,
• Exacerbation of a pre-existing illness,
• Increase in frequency or intensity of a pre-existing episodic event or condition,
• Condition detected or diagnosed after intervention, even though it may have been present prior to the start of the study, and
• Continuous or persistent disease or symptoms present at baseline that worsen following the start of the study.

The AE were collected in a separate database with the complete description of the event. If the AE was related to the intervention, it had been reported to the ethics committee.

### Sample Size

Sample size was determined using the existing literature. Two exercise interventions were evaluated using a pilot study in individuals suffering from AD. Sample sizes were 7 and twelve participants.^40,68^

### Statistical Methods

Normality of the data was assessed with the Shapiro–Wilk test. For primary and secondary outcomes, the mean difference between initial and final evaluation and the 95% confidence interval (95% CI) were calculated using Student’s*t* test for paired samples. The Wilcoxon signed-rank test was used to identify change significance for not normally distributed variables. Significance level was established at *P* < .05. Effect sizes were calculated using Cohens’ d, with .2 indicating a small effect, .5 a moderate, and .8 a large effect.^69^

To enable clinical interpretation, minimal clinically important differences (MCID) or minimal detectable differences (MDC) were used for comparison of the primary outcomes. If none was available for a population suffering from AD or cognitive impairment, the value for the most similar population was considered.^70^ BBS MCID for a person suffering from multiple sclerosis is 3 points.^71^ MDC_95_ for TUG (2.42 seconds)^55^ and FTSTS (2.5 seconds)^52^ were used. MDC of CWS in adults with AD is .09 meters/second (m/s).^50^ MDC for MWS is .19 m/s in stroke patients with a comfortable walking speed ˃0.8 m/s.^72^

MCID or MDC for SCWT and TMT-B were only available in seconds and therefore not comparable with the scores from our analyses.^73,74^ DST-B evaluations were not acceptable for evaluation of MCID or MDC.^75^

Primary and secondary outcomes were evaluated using R version 4.3.0.^76^ Feasibility outcomes were assessed using both R version 4.3.0 and Microsoft Excel.

## Results

No changes to the protocol were made after the start of the study.

### Participants

**Figure 3.**
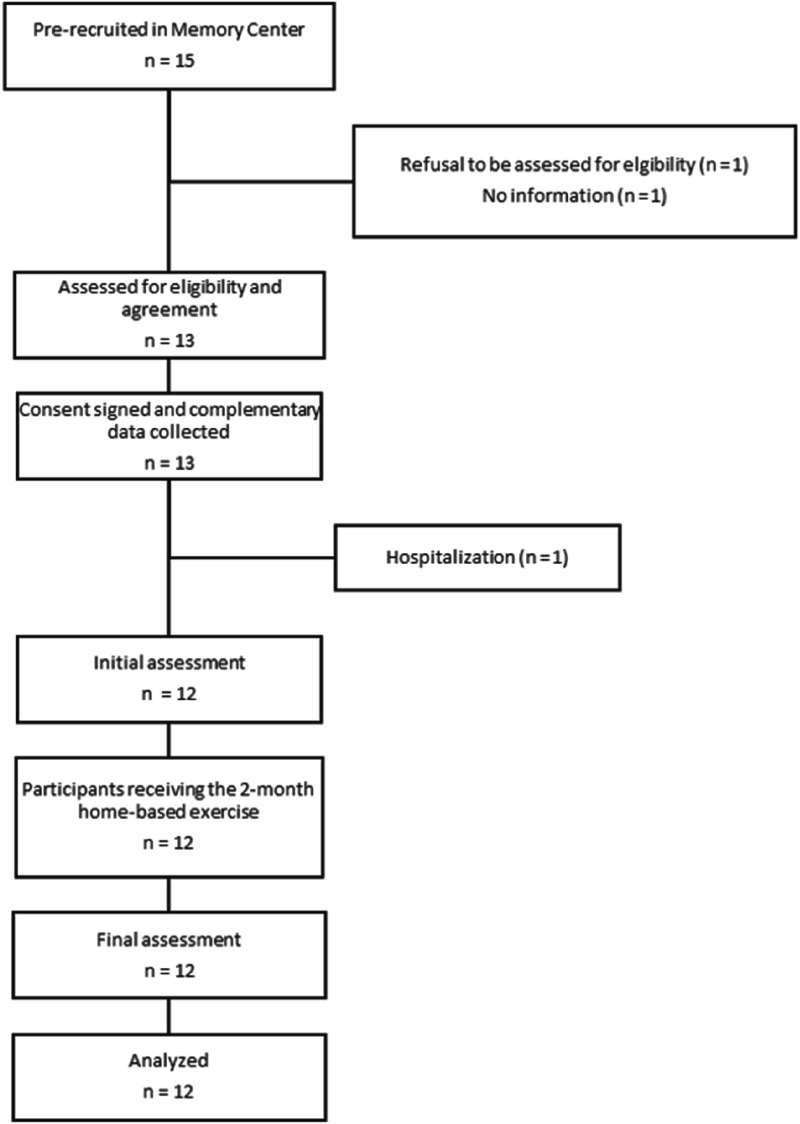
Flow chart of the participants.

Between June 30 and December 6, 2021, 15 patients were recruited at the Memory Center of Valais Hospital in Sierre, of which 13 were eligible. One patient was hospitalized between the first meeting and the initial evaluation and did not begin the study. The 12 included participants continued until the final evaluation (Figure 3). The interventions occurred between August 2021 and March 2022.

Table 1 describes the baseline characteristics of the participants. Furthermore, all participants had at least 1 comorbidity and took an average of 7.75 drugs per day.

**Table 1.** Baseline Characteristics of the Participant.

		Total Sample (n = 12)
Age, mean ± SD		80.83 ± 4.65
Gender, n (%)		
	Male	5 (41.67)
	Female	7 (58.33)
Higher education level, n (%)		
	Compulsory school	11 (91.67)
	University degree	1 (8.33)
Using a walking aid, n (%)		4 (33.33)
Stage of Alzheimer disease, n, (%)		
	Early stage	2 (16.67)
	Prodromal stage	10 (83.33)
SAFER-HOME, median (range)		4 (0 to 32)
EQ-5D-5 L, mean ± SD		.70 ± .26
Depression score, mean ± SD		3.50 ± 2.07
Score of quality of life, mean ± SD		37.33 ± 3.58

Although all the participants were accompanied by a related caregiver during the consultation at the memory clinic, 5 caregivers declined to participate in the exercises at home. The reasons were either that the husband or spouse was not interested or that the children or grandchildren lived too far away.

Regarding completion of the weekly diaries, 2 participants never completed their diaries; however, 1 told the physiotherapist that he had trained regularly, and the second rarely. Another participant wrote only once in his weekly diary but told the physiotherapist that she had trained regularly without documenting it.

One participant admitted to training only once a week. One participant had a change in condition toward the end of the study with a decline in his health condition.

During the weekly sessions with the physiotherapist, all participants used the tablet, but only 4 of them used the tablet with their caregiver outside the physiotherapy sessions; however, 2 participants used the tablet independently throughout the study.

## Primary Outcomes

All the basic functional mobility measures showed small effect sizes (Cohen’s d between .20 and .42) and improved between the initial (baseline) and final evaluation (week 9); however, only the FTSTS mean difference (−2.58; 95% CI -6.52 to 1.37) in seconds reached a clinically meaningful change (MDC_95_ 2.5 seconds). None of the mean differences between pre- and post-intervention evaluations were statistically significant. Scores at baseline and final evaluations, as well as the mean differences between both evaluations are displayed in Table 2.

**Table 2.** Primary Outcomes Measures (n = 12).

Test	Baseline *Mean (95% CI)*	Final Evaluation *Mean (95%CI)*	Mean Difference (95% CI)	*P*-value	Effect Size *Cohen’s d*
Berg balance scale score	49.67 (45.16 to 54.17)	51.08 (47.38 to 54.78)	1.42 (−1.03 to 3.86)	.229	.37
Timed up and Go (sec)	14.14 (10.32 to 17.96)	13.53 (9.25 to 17.80)	−.61 (−2.40 to 1.18)	.470	.22
Five time sit to stand (sec)	17.77 (12.96 to 22.59)	15.20 (10.67 to 19.72)	−2.58 (−6.52 to 1.37)	.178	.42
Comfortable gait speed (m/s)	.91 (.73 to 1.09)	.95 (.75 to 1.14)	.04 (−.08 to .15)	.503	.20
Maximal gait speed (m/s)	1.23 (.99 to 1.47)	1.27 (1.02 to 1.52)	.04 (−.04 to .13)	.266	.34

n: number of participants; 95% CI: 95% confidence interval; sec: second; m/s: meters per second.

## Secondary Outcomes

Four outcome measures assessing EF showed small effect sizes (Cohen’s d between .03 to .40), and 3 a moderate effect size (Cohen’s d .52 to .54). Most measures improved between the 2 evaluation timepoints; however, DST-B and SCWT-C scores worsened. Performance classification of SWCT remained stable at 2 (moderate), but for panel W, it increased from limit to moderate (1.5 to 2). None of these changes were statistically significant. Scores of the secondary outcome measures are displayed in Table 3.

**Table 3.** Secondary Outcome Measures (n = 12).

Test	Baseline	Final Evaluation	Difference	*P*-value	Effect Size*Cohen’s d*
Trail making Test-Be in metric^a^	14.09 (8.70 to 19.48)	13.27 (7.65 to 18.89)	−.82 (−2.61 to .97)	.337	.29
Digit Span backward Task^a^	4.92 (3.96 to 5.87)	3.92 (2.92 to 4.91)	−1 (−2.21 to .21)	.097	.52
Stroop color-word test					
Colour, Z-score^a*^	−.75 (−1.77 to .28)	−.78 (−1.62 to .07)	−.03 (−.62 to .56)	.519	.03
Colour, performance^b^	2 (.75 to 2)	2 (.75 to 2)			
Word, Z-score^a^	−1.57 (−2.92 to −.21)	−1.08 (−2.15 to −.02)	.48 (−.30 to 1.26)	.203	.39
Word, performance^b^	1.5 (0 to 2)	2 (.75 to 2)			
Interference, Z-score^a^	−1.12 (−3.03 to .79)	−.46 (−1.68 to .77)	.66 (−.39 to 1.71)	.193	.40
Interference, performance^b^	2 (0 to 2.25)	2 (0 to 3)			
Interference index if, Z-score^a^	−.08 (−.87 to .71)	.44 (−.59 to 1.47)	.52 (−.10 to 1.14)	.093	.53
Interference index if, performance^b^	2 (1.75 to 2)	2 (1 to 3)			
Interference index IF, Z-score^a*^	−.51 (−2.05 to 1.03)	.50 (−.38 to 1.39)	1.01 (−.09 to 2.12)	.092	.54
Interference index IF, performance^b^	2 (.75 to 2.25)	2 (1 to 3)			

^a^Mean (95% confidence interval).

^b^Median (interquartile range); n: number of participants; *baseline and final results, as well as mean differences are presented as the mean (95% confidence interval), using *t* test despite nonnormal distribution to enable comparison, but *P*-values were calculated using Wilcoxon signed-rank test.

## Feasibility

Three out of 4 feasibility criteria were met, only the cost adequacy criterion was slightly above the pre-set threshold.

### Recruitment Opportunity

The memory clinic of the Hôpital du Valais in Sierre was able to recruit 15 participants between the middle of week 26 and the beginning of week 49 of 2021, i.e in total less than 23 weeks. This was below the fixed threshold of 24 weeks.

### Participation Agreement Rate

13 out of 13 eligible participants agreed to participate. This criterion has been positively exceeded.

### Cost adequacy

The total costs of this pilot study were CHF 95 200.-, slightly above the planned budget. Costs were allocated as follows: CHF 17 300.- for the physiotherapy sessions, CHF 76 700.- for the study personal and CHF 1200.- for the other costs.

### Drop Out/Withdrawal Rate

The only dropout occurred between the signature of the informed consent and the initial assessment. No dropouts occurred between initial and final assessment, the dropout/withdrawal rate is therefore 0% and fully met the set criterion.

### Adverse Events

One participant with a kidney transplant had to return to dialysis at the end of study participation. Despite this, he participated until the final evaluation. No participant was harmed during the study.

## Discussion

The purpose of this exploratory single-group pilot study using a pre- and post-test design was to assess the feasibility of a study in persons with prodromal or mild AD using an adapted T&E home-based exercise program and its effectiveness on basic functional mobility and EF. The 5 measures of basic functional mobility and 2 of 3 measures of EF showed a trend towards improvement, without statistical significance.

In older people with cognitive impairment or dementia, supervised physical exercises for about 60 minutes, two to 3 days a week, may improve mobility or functional limitations.^77,78^ In this study, the recommendations were to train 3 times a week for 30 to 45 minutes, once under supervision of a physiotherapist and twice independently, with or without their caregiver. The participants who improved their scores on basic mobility assessments were those who trained regularly between the sessions with the physiotherapist.

Mental flexibility, evaluated with the TMT-Be, and response inhibition, evaluated with the SCWT, showed a trend toward improvement, but the update evaluated with the DSTB showed a decrease. Although the results of this pilot study were not statistically significant, they were consistent with some of the results in Arcoverde et al^79^ who also found an improvement in TMT-A, but a deterioration in DST-B. Huang et al^80^ also assessed the effect of home-based exercise programs (resistance or aerobic training) in people suffering from prodromal AD or MCI. DST-B was stable or slightly increased. The effect on SCWT depended on the panel and differed between the 2 groups. Liu-Ambrose et al also sought to assess the effects of a home-based exercise program on cognitive function, but in healthy older people. The time to carry out TMT-B and SWCT improved after 6 months, while the DST-B score remained stable. The values of DST-B (3.8 ± 2.0 to 3.9 ± 2.3) were lower than those in the current pilot study (4.92 [95% CI 3.96-5.87] to 3.92 [95% CI 2.92-4.91]).^81^

Although all participants were accompanied by a caregiver during their visit to the hospital's memory clinic, only seven caregivers agreed to participate in the study. While physical exercise is recommended for people with AD, the presence of a caregiver may enhance the benefits of exercise in this population.^82,83^

Participants had the choice of using an Android application on a tablet, and/or a booklet and cards for exercising. The ability to use the tablet independently in the current pilot study was lower than stated in the review of Joddrell & Astell^84^ where 1 half to two-thirds of the participants were capable of independent use. In Switzerland, the use of touch-screen devices among the elderly population is steadily increasing; however, their use is higher in the youngest older people. Furthermore, over 50% of those aged 65+ use a smartphone daily, but less than 30% use a tablet.^85^ The lack of computer literacy in the elderly and the perceived difficulty of the tool may be barriers to the use of tablets.^86^

Given the feasibility criteria alone, a future randomized clinical trial to assess the effects of a T&E home-based exercise program for older people with MCI or early AD may be possible; however, there are several considerations. First, with regards to the sample, the results of this pilot study can be used to determine the sample size. Furthermore, the importance of social and health networks for recruitment will need to be carefully considered. Alzheimer’s disease not only causes suffering to patients themselves but also to their relatives. An AD diagnosis has psychological, physical, and well-being impacts on family caregivers.^87,88^ It is therefore even more important to work closely with recruitment centers and to have very empathetic recruiters at the beginning of the study. Second, the choice of measurement tools was appropriate and corresponded to the current recommendations for this population^36^; however, an assessment of activities of daily living (ADL) should also be carried out. As recommendations, the Bristol ADL^89^ or the Disability Assessment for Dementia^90^ could be used.^91^ It would also be of interest to assess the cognitive state of the participants at the beginning of the study with the mini-mental state evaluation (MMSE85) or the Montreal cognitive assessment (MoCA).^92^ Both assessments are effective tools to detect AD^93^; however, MoCA enables better detection of MCI.^94^ Such information would enable subgroup analyses to be performed and to determine whether the program might have different benefits according to the baseline cognitive level. Third, costs should be carefully planned, and a greater allowance should be made for personnel costs. Listing every major and minor task and a realistic time allocated for them is a key factor for planning a representative budget.^95^

The 2 main limitations of this study were the small sample size and the lack of a control group; however, further research and larger studies in this field are needed.

In conclusion, the home-based exercise program T&E may be effective for patients suffering from AD; however, no clinical recommendations can be made based on the results of this study even though they were in line with the current literature. Although all feasibility criteria were mostly met and a trend toward improvement in the primary and secondary outcomes was shown, further research on the use, ability to use and preferences in digital technologies among people suffering from prodromal or mild AD is needed before carrying out a future larger-scale study on this subject.

## Data Availability

The datasets generated during and analyzed during the current study are available from the corresponding author on reasonable request.*

## Appendix

### Abbreviations

AD: Alzheimer’s disease
ADL: activities of daily living
AE: adverse event
BBS: Berg Balance Scale
CHF: Swiss Francs
CI: Confidence interval
CWS: comfortable walking speed
DST: Digit Span Test
DST-B: Digit Span Test Backward
DST-F: Digit Span Test Forward
EF: executive functions
EQ-5D-5 L: EuroQoL-five dimensions-five levels
FTSTS: five time sit to stand
GDS-15: geriatric depression scale
ICC: intraclass correlation
MCI: mild cognitive impairment
MCID: minimal clinical important differences
MDC: minimal detectable difference
MMSE: Mini-Mental State Examination
MoCA: Montreal cognitive assessment
MWS: maximal walking speed
QOL-AD: Quality Of Life questionnaire for Alzheimer’s Disease
REDCap: Research Electronic Data Capture
SAFER-HOME: Safety Assessment of Function and the Environment for Rehabilitation – Health Outcome Measurement and Evaluation
SCWT: Stroop Color-Word test – Victoria
SCWT-C: Stroop Color-Word test – Victoria - Panel Color
SCWT-I: Stroop Color-Word test – Victoria – Panel Interference
SCWT-W: Stroop Color-Word test – Victoria – Panel Word
SCWT-if: Stroop Color-Word test – Victoria – weak interference index
SCWT-IF: Stroop Color-Word test – Victoria – strong interference index
T&E: Test-and-Exercise
TMT-B: Trail Marking Test Part-B
TMT-Be: Trail Marking Test Part-B efficiency
TMT-Bs: Trail Marking Test Part-B in seconds
TUG: Time Up and Go Test
